# Recent Advances on Octahedral Polypyridyl Ruthenium(II) Complexes as Antimicrobial Agents

**DOI:** 10.3390/polym10060650

**Published:** 2018-06-10

**Authors:** Yulin Yang, Guojian Liao, Chen Fu

**Affiliations:** College of Pharmaceutical Sciences, Southwest University, Chongqing 400715, China; yyl1996@email.swu.edu.cn (Y.Y.); gjliao@swu.edu.cn (G.L.)

**Keywords:** polypyridyl, octahedral, ruthenium complex, antimicrobial activities

## Abstract

Recent developments of therapeutic agents based on transition metals have attracted a great deal of attention. Metal drugs have advantages over other small molecule drugs, and it was demonstrated that, in a number of studies, they played an important role in pharmaceutical chemical research and clinical chemotherapy of cancers. It is worthwhile mentioning that octahedral polypyridyl ruthenium(II) complexes have shown remarkable applications in chemical biology and medicinal chemistry over the last decade. However, only very recently has there been comprehensive interest in their antimicrobial properties due to metal-related toxic concerns or neglected potential roles in microbiological systems. Our review will highlight the recent developments in octahedral polypyridyl ruthenium(III) complexes that have exhibited significant antimicrobial activities and will discuss the relationship between the chemical structure and biological process of ruthenium complexes, in both bacterial and fungal cells.

## 1. Introduction

Bacterial resistance is a major challenge for the global public health community [1,2]. In recent years, various types of antibiotic and antibacterial drug-resistant bacteria have developed rapidly. Methicillin-resistant *Staphylococcus aureus* (MRSA), penicillin-resistant *Streptococcus pneumoniae* (PRSP), vancomycin-resistant *Enterococcus* (VRE), etc., have caused serious difficulties in clinical treatment [3]. Therefore, tremendous research efforts have been devoted to the development of novel and potent antimicrobial agents for the past decades [4,5].

By and large, there are three strategies for the control of drug-resistant bacterial infections and the exploration of anti-drug resistance drugs. (A) The development of antimicrobial auxiliary agents. Along with more intimate knowledge of antibacterial drugs themselves, many research institutes have now shifted their focus to these study of auxiliary agents, which have shown synergistic and regulatory effects on antibacterial agents [6]. (B) The structural modification of existing antibiotics or synthetic antimicrobials under the guidance of research achievements in the mechanisms of action, resistance mechanisms, structure–activity relationships, etc. [7]. (C) The study of novel antibacterial agents based on novel chemical structures, mechanisms, and targets of action, which are effective approaches to overcome the resistance of pathogenic bacteria [8].

Metal-based drugs play a significant role in the history of medicinal chemistry [9]. Several excellent reviews describing novel transition metal drugs have shown the advantages over traditional organic molecules, such as ready structural modification, rich photo-physical and electrochemical properties, etc. [10,11,12,13,14]. To date, many transition metal complexes have been reported to have a number of excellent biopharmaceutical activities, and many of them have been applied in clinical practice. Basically, metallic drug research covers many aspects, such as anti-cancer drugs, anti-diabetic drugs, anti-parasitic drugs, and anti-bacterial drugs [15]. Among others, the development of anti-cancer drugs is currently the main area of concern, and major advances have been made in the rational design of drugs [16].

Ruthenium and ruthenium complexes are easily absorbed by tumor tissues and are excreted in vivo quickly, in addition, they possess low toxicity and have good anticancer application prospects [17]. Compared with small molecule drugs, ruthenium complexes can provide a unique modular system [18]. The centrally-located ruthenium atom actually acts as a structural center, supporting a rigid, three-dimensional scaffold of ligands, which could lead to a large number of ruthenium complexes with different structural properties via facile ligand substitution or modification, including simple chemical modification, combination of complexes with small organic molecules, introduction of chiral groups, linkage of complexes with macromolecule polymers, matching complexes with specific receptors of cancer cells or other anti-cancer drugs, combination of complexes and groups with special functions, such as photosensitivity, thermosensitivity, and so on [19,20]. Using these methods, not only new complexes with significant anti-cancer activities could be obtained, but also some predesigned physicochemical properties of these complexes, such as water solubility, lipid solubility, and targeting properties could be precisely achieved [21].

Polypyridyl complexes are coordination compounds in which a polypyridine, such as 2,2′-bipyridine (bpy) or 1,10-phenanthroline (phen), coordinates to the centered metal ion or atom [22]. A well-known example of polypyridyl complexes is tris(2,2′-bipyridine) ruthenium(II) complex, Ru[(bpy)_3_]^2+^, the derivatives of which are numerous and widely utilized in the areas of photoredox catalysis and life sciences [23,24]. The complexes are reasonably stable to light, electricity and heat, because the bonds between the central metal and polypyridyl ligands are very strong. These complexes possess specific optical, electrochemical properties; moreover, some of these exhibit a strong absorption band in the visible light range, namely the metal-to-ligand charge transfer (MLCT) [25]. The MLCT absorption band, emission wavelength, and emission lifetime can be easily varied by the introduction of various substituents with different electronic properties to the polypyridyl ligands. It has also been confirmed by considerable studies that polypyridyl ruthenium(II) complexes are potential chemotherapeutic drugs that can bind to various nucleic acid sequences in different modes, such as insertion and groove cross-binding [26,27]. Thus, the complexes can be used as specific inhibitors for telomerase, DNA topoisomerase, protein kinase, and so on, to regulate cell pathways and induce tumor cell apoptosis [28,29,30,31].

## 2. Ruthenium-Based Microbiological Activities and Antimicrobial Mechanisms

Since the pioneering studies from the Barton group on the design of substitutionally inert ruthenium complexes as efficiently selective DNA binders [32], octahedral polypyridyl ruthenium(II) complexes have been intensively investigated and have shown remarkable applications in chemical biology and medicinal chemistry over the last few decades [33,34]. Contrary to the well-established anticancer drug cisplatin, metal complexes merely play a structural role in most cases. Hence, there are some obvious advantages for octahedral ruthenium complexes over common organic molecules, serving as a structural scaffold for the development of bioactive molecule inhibitors [35,36].

In general, according to the suggested categorization for metal anticancer compounds, antimicrobial ruthenium(II) complexes can also be divided into five groups, based on their mode of action [37].

(A) Functional role: The Ru metal center should bind to the biological target, i.e., the antimicrobial activity of the complex stems from the coordination of the metal center with biological targets. Similar to cisplatin, these complexes should bear labile ligands, which can be dissociated and leave coordinatively-unsaturated intermediates [38].
(B) Structural role: The Ru metal acts as a structural center, i.e., the centered metal is purely employed as a structural octahedral center, as in many metallo-intercalators or insertors [39]. These kinds of complexes are kinetically inert and biologically stable; the interaction of these complexes with the biological targets only occurs through non-covalent interactions.
(C) Carrier: The metal center acts as a carrier for active ligands, normally-established drugs, to enhance their pharmaceutical activities via temporary coordination with the metal moiety [40].
(D) Catalyst: The metal complex behaves as a catalyst, i.e., the substitutionally-inert complex can catalyze a redox cycle for the oxidation of glutathione (GSH) to glutathione disulfide (GSSG), which will lead to an obvious increase in reactive oxygen species (ROS) and is highly cytotoxic against some cell lines [41].
(E) Photo-sensitizer: The metal complex is photo-active and can be used as a photo-sensitizer, i.e., polypyridyl Ru(II) complexes retain the capability to produce singlet oxygen because of their low-energy triplet excited state [42].

Up to now, hundreds of ruthenium-based complexes have been reported in antimicrobial studies [43,44,45]. In fact, the first ruthenium antimicrobial agent dates back to around sixty years ago [46]. However, only very recently has there been a comprehensive interest in their antimicrobial properties due to metal-related toxic concerns or neglected potential roles in microbiological systems. The antimicrobial mechanisms of octahedral ruthenium complex-DNA interactions have been extensively researched, and, in this text, we review that some examples of ruthenium complexes interact with DNA in its various structural forms, using various different binding modes. Based on the classification described above, the role of the ruthenium centers in the reported antimicrobial complexes is just to work as a structural scaffold for all bioactive ligands surrounding the centered metal (in most cases). The ruthenium metal center does not directly interact with the biological targets, but rather via non-covalent interactions [47].

As we know, no example of a ruthenium complex inhibiting a specific enzyme in live bacteria has been reported. Nevertheless, it is generally believed that proteins and enzymes could still be promising targets for the development of octahedral polypyridyl ruthenium complexes as potent antimicrobial agents, under sufficient consideration of elaborate stereochemistry of octahedral coordination geometries and reasonably modified lipophilicity [48].

## 3. Polypyridyl Ruthenium(II) Complexes with Different Structures

**Figure 1:**
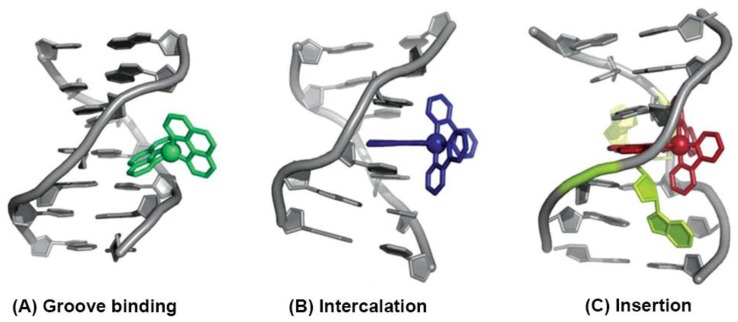
The three binding modes of metal complexes with B-DNA: (**A**) groove binding, (**B**) intercalation, and (**C**) insertion (reproduced with permission from Reference [18]).

Recently, the biological properties of kinetically-inert polypyridyl ruthenium(II) complexes have attracted growing interest [49]. These hexacoordinated ruthenium(II) complexes are capable of reversibly interacting with important biomolecules, including DNA, RNA, proteins, etc. [19]. As these inert complexes remain stable in vivo, they can reversibly bind with DNA, RNA, or proteins via noncovalent bonds, such as groove binding, intercalation, or insertion, as shown in Figure 1. This type of binding mode could lead to various biological responses, for instance, if the binding affinity of the metal complex with the target is high, then the dissociation rate from the biological target could be fairly low; structural distortion by the complex may cause permanent dysfunction of the target leading to bacteriostasis or cell death. Moreover, for an octahedral complex with six different monodentate ligands, the number of related stereoisomers that can be achieved may be up to 30 [50]. Most polypyridyl ruthenium(II) complexes are chiral and they can, accordingly, display bioactive differences in the process of binding to chiral biological receptors. As a result, the elaborate stereochemistry of an octahedral ruthenium(II) complex is, nowadays, a key feature of many applications in different areas of life sciences [51].

### 3.1. Mononuclear Polypyridyl Ruthenium Complexes

In 1952, Dwyer et al. reported their pioneering antimicrobial study of these kinetically-inert ruthenium complexes, and they studied the bioactivities of the simplest polypyridyl ruthenium(II) complexes—mononuclear metal complexes with three identical bidentate ligands, such as bpy, phen, and their derivatives [46]. The antibacterial activities of these complexes were examined against different bacterial cells, including Gram-positive, Gram-negative, and acid-fast bacteria. The results showed that [Ru(phen)_3_]^2+^ was inactive against all the bacterial strains, although the introduction of the methyl group on the phen ligands remarkably enhanced its activity against all tested bacteria, which could be attributed to the increase in lipophilicity [24].

Unfortunately, the groundbreaking results did not boost further developments of the mononuclear polypyridyl metal complexes as therapeutic agents, which could be attributed to two aspects: One is that a great deal of attention has been generated by their fantastic nucleotide affinity since then, and the other is that people at that time were more interested in antibiotics. However, recently, there has been renewed concern regarding the antimicrobial activities of polypyridyl octahedral ruthenium(II) complexes. Aldrich-Wright et al. found that the mononuclear ruthenium complex [Ru(2,9-Me_2_phen)_2_(dppz)]^2+^ showed remarkable bactericidal activity against *S. aureus* and *Bacillus subtilis* strains, and it also could bind with DNA via intercalation [31]. The antibacterial activity of the tested ruthenium complexes was in the order of dppz>dpqC>dpq, which also coincides with the order of their DNA-binding affinity. The complex with the dppz ligand possesses the highest affinity, in contrast to the complex that has the lowest affinity with ligand dpq. In addition to in vitro studies, the authors also reported that [Ru(2,9-Me_2_phen)_2_(dppz)]^2+^ was active in vivo because it prevented the death of *Caenorhabditis elegans* nematodes infected with *S. aureus*. More importantly, the complex was verified as nontoxic to *C. elegans*, suggesting that they were nontoxic against eukaryotic systems as well.

**Figure 2:**
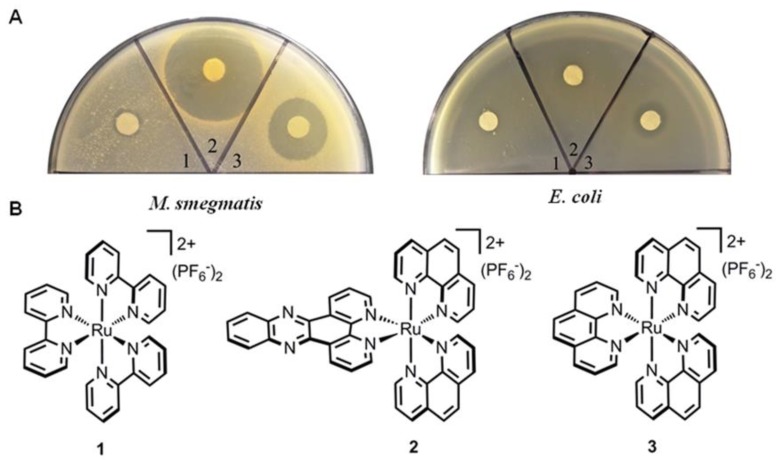
Antimicrobial activities of octahedral ruthenium complexes. (**A**) Strains including *E. coli* ATCC25922 and *M. smegmatis* mc2155 were used as indicator strains; (**B**) chemical structures of complexes (**1**–**3**) (reproduced from Reference [52]).

Recently, our group discovered that two typical octahedral ruthenium(II) complexes could selectively inhibit the growth of *Mycobacterium smegmatis* (Figure 2) [52]. The results revealed that complex **2** [(phen)_2_Ru(dppz)](PF_6_)_2_ (dppz = dipyridophenazine) could notably inhibit the growth of *M. smegmatis* with the minimum inhibitory concentration (MIC) value of 2 μg/mL, while complex **3** [Ru(phen)_3_](PF_6_)_2_ was bactericidal with an MIC value of 26 μg/mL. In addition, these two complexes should possess different antimicrobial mechanisms, because the bactericidal activity of [Ru(phen)_3_](PF_6_)_2_ was dependent on reactive oxygen species (ROS) production. Furthermore, both complexes showed no cytotoxicity against LO2 and hepG2 cell lines at concentrations as high as 64 μg/mL. These findings suggest that ruthenium complexes could serve as a useful lead compound for further modification to explore novel antibacterial agents for the treatment of *M. tuberculosis* infection.

**Figure 3:**
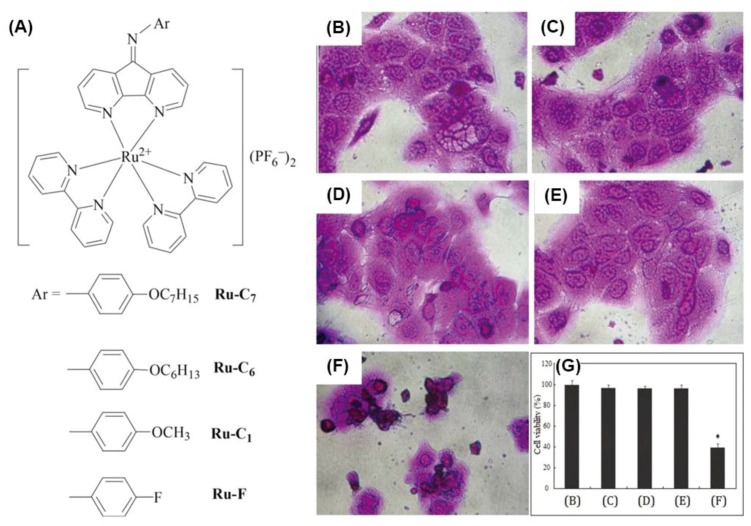
(**A**) Chemical structures of new ruthenium(II) complexes containing various *N*-phenyl-substituted 4,5-diazafluorenes;cytotoxicity evaluation of the ruthenium(II) complexes on human skin keratinocytes: (**B**) untreated control; (**C**) vehicle control (0.1% DMSO); (**D**) complex Ru-C6 (50 μg/mL); (**E**) complex Ru-C7 (50 μg/mL); (**F**) doxorubicin (2 μg/mL); and (**G**) cell viability of ruthenium(II) complexes towards human skin keratinocytes. Each experiment was carried out in triplicate and a mean value was obtained. Results were determined from three independent experiments and standard deviations are provided (reproduced with permission from Reference [53]).

For the antimicrobial activities of polypyridyl octahedral ruthenium(II) complexes, a massive number of findings have suggested that DNA binding is a logical candidate in terms of bioactive mechanisms. As shown in Figure 3, Wong et al. synthesized a series of bis-bpy ruthenium(II) complexes harboring *N*-phenyl-substituted diazafluorenes (Ru-C1, Ru-C6, Ru-C7 and Ru-F) and investigated their potential antibacterial activities against MRSA [53]. Compared to the control of methicillin {MIC = 25μg/mL; MBC (minimum bactericidal concetration) = 100 μg/mL}, Ru-C7 exhibited notable improvement in both MIC value (6.25 μg/mL) and MBC value (25 μg/mL) against MRSA, which could be due to DNA damage caused by ROS. In addition, Ru-C7 possessed much stronger antibacterial activities than Ru-C6 (MIC = 25 μg/mL, MBC = 100 μg/mL), while the two complexes were also proved to be biologically safe when examined on normal human skin keratinocytes (more than 90% of skin cells survived in the presence of ruthenium complexes at a concentration of 50 μg/mL).

Polypyridyl ruthenium(II) complexes with specific photophysical properties could achieve their microbiological effects via photodynamic antimicrobial chemotherapy (PACT). Donnelly and coworkers described, for the first time, that ruthenium(II) complex ([Ru(dmob)_3_]Cl_2_), with dmob = 4,4′-dimethoxy-2,2′-bipyrdine, could serve as a photosensitiser for use in PACT [54]. As a result, the photosensitizer exhibited toxic effects on microorganisms that could be intensified remarkably via irradiation. Moreover, it was the most effective photosensitizer tested, especially when irradiated, with the prevention of growth for all three isolates which could be killed in a concentration of 50 μg/mL.

### 3.2. Dinuclear Polypyridyl Ruthenium Complexes

Few dinuclear or oligonuclear polypyridyl octahedral ruthenium(II) complexes have been extensively studied for their antimicrobial activities until recent years [55]. These dinuclear complexes are the analogs of the mononuclear complex moieties bridged by a flexible linker ligand. Compared to the corresponding mononuclear complexes, the DNA binding capabilities of dinuclear complexes have been improved significantly. Dinuclear ruthenium(II) complexes usually possess notable bactericidal activities against a wide range of Gram-positive and Gram-negative bacteria, including drug susceptible strains and clinical isolates [34]. Ruthenium complexes containing commonly-used bridging ligands, such as dppz and tpphz ligands, have also been determined to exhibit a high affinity to duplex or G-quadruplex DNA by intercalation with low salt concentration dependence.

**Figure 4:**
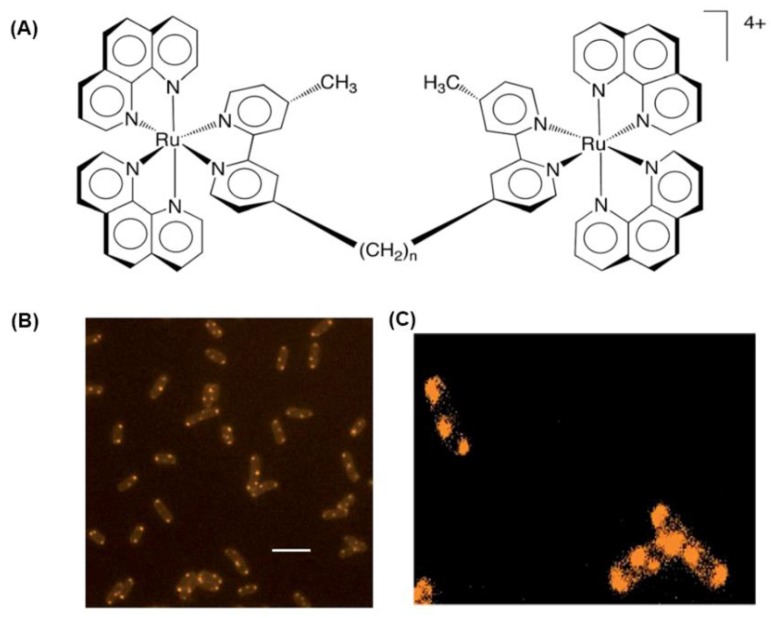
(**A**) The structure of the dinuclear polypyridyl ruthenium(II) complexes Rubbn, where *n* = 2, 5, 7, 10, 12 and 16; (**B**) phosphorescence fluorescence microscopy images of Rubb16 localization in *E. coli* MG1665 cells at MIC concentration (4 mg/mL), scale bar = 5 μm; (**C**) Rubb16 localization in *E. coli* MG1665 cells at twice the MIC concentration with the image re-processed to enhance the luminescence of the Rubb16 outside of the foci (reproduced from Reference [56]).

Keene and Collins recently demonstrated a series of dinuclear ruthenium(II) complexes that possess a flexible CH_2_– chain as the bridging ligand, which showed significant antimicrobial activities against both Gram-positive and Gram-negative bacteria, as well as against MRSA (Figure 4) [56]. They used wide field fluorescence microscopy to investigate the intracellular binding site of Rubb16 in *Escherichia coli*. Rubb16 localized at the ribosomes with no obvious DNA binding on the basis of the incubation of *E. coli* cells under the MIC concentration. In addition, it condensed the ribosomes when they existed as polysomes, which would terminate the production of proteins and inhibit bacterial growth accordingly. Moreover, the Rubbn complexes are significantly less toxic to red blood cells and a human leukemia cell lines than against bacteria [57]. The results of this research suggest that the family of inert polypyridyl dinuclear ruthenium complexes could selectively target RNA over DNA in vivo. Owing to the differences in ribosome structure between bacteria and eukaryotic cells, selective RNA targeting could be favorable for the development of therapeutic agents.

### 3.3. Multinuclear Polypyridyl Ruthenium Complexes

**Figure 5:**
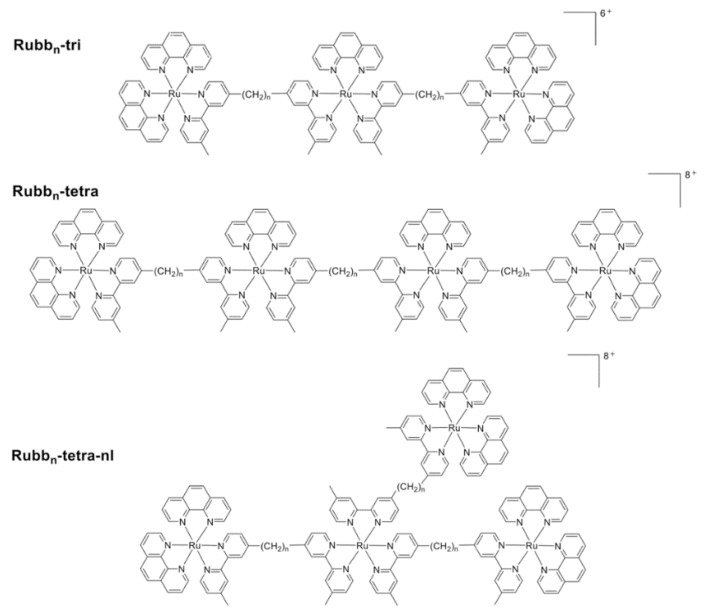
Chemical structures of the trinuclear polypyridyl ruthenium(II) complexes (Rubbn-tri), linear tetranuclear complexes (Rubbn-tetra) and nonlinear multinuclear ruthenium(II) complexes (Rubbn-tetra-nl)(reproduced from Reference [59]).

Studies on the antimicrobial activity of corresponding tri- and tetra-nuclear complexes have also been carried out, considering that proper antimicrobial activities have been achieved by the dinuclear Rubbn complexes [58]. In addition, based on the modular nature of these metal complexes, facile syntheses of polypyridyl ruthenium(II) complexes with different configurations, for instance, linear and non-linear structural shapes, could be realized. It has been reported that all the tri- and tetra-nuclear complexes displayed ideal antimicrobial activity, examples include, the most active compounds, such as linear Rubb12-tri, Rubb16-tri, Rubb12-tetra, and Rubb16-tetra, exhibited up to four-times more activity than the related dinuclear complexes [57]. Based on the lipid–water distribution coefficient (log *p* values), trinuclear ruthenium(II) complexes possessed the most lipophilic properties, however, the linear tetranuclear complexes were generally more active (Figure 5). The level of cellular accumulation of tri- and tetra-nuclear ruthenium(II) complexes in Gram-negative bacteria was uniform to, or greater than, that in Gram-positive species. Notably, a lower activity was observed against Gram-negative species, which suggested that some Gram-negative species, particularly *Pseudomonas aeruginosa*, have an inherent resistance to these polypyridyl ruthenium(II) complexes [59].

## 4. Conclusions

It is known that irrational application, or even abuse, of antibiotics has resulted in increasingly serious drug resistance, which has also become one of the most pressing public health challenges around the world. The development of novel and efficient antibacterial drugs with new chemical structures and mechanisms has been recognized as the main approach to solve drug resistance problems. Due to the diversity of geometrical structures, metal complexes have a richer steric chemistry than ordinary organic molecules, and it is more convenient for metal complexes to introduce chiral moieties as well, which would benefit the specific recognition of biomolecules. Similarly, metal complexes could have a high positive charge, and many biomolecules, such as DNA, RNA, some phospholipid molecules, and some proteins, have a negative charge; therefore, metal complexes can bind with these intracellular biological targets based on the existence of electrostatic attraction. A large number of studies have indicated that a great number of transition metal complexes can bind specifically to DNA or RNA, and show anti-cancer activities, some of which have also exhibited substantial antibacterial activities [60].

The application of platinum complexes, such as cisplatin and carboplatin, has been very successful in clinical applications, but there are still some non-negligible disadvantages to these metal complexes, including excessive cytotoxicity and great side effects. As a result, people turned to other transition metals for drug replacement. Sixty years ago, Dwyer et al. found that ruthenium complexes had certain antibacterial activities but owing to the high tide of the development of organic antibacterial agents at that time, unfortunately, these metal complexes did not receive sufficient attention. In recent years, ruthenium complexes have regained a great deal of attention with the increasing demand for new antibiotics.

Inert polypyridyl ruthenium(II) complexes and their derivatives have achieved notable important advances in the fields of anti-cancer and anti-bacterial activities. A number of highly bioactive metal complexes have been discovered through ligand substitutions and modifications. The investigation of the structure–activity relationship and the discovery of active group positioning rules have laid a solid foundation for further research and development of inert polypyridyl ruthenium(II) complexes as antibacterial drugs. However, it should be pointed out that the mechanisms of bactericidal action of polypyridyl ruthenium(II) complexes have not been studied thoroughly. A great deal of work thus far has only focused on the study of DNA interactions, and the reasons why some complexes could highly selectively inhibit bacterial proliferation, and even kill bacteria, have rarely been reported. Overall, research on the antibacterial activities of polypyridyl ruthenium(II) complexes have become a new hot topic. Polypyridyl ruthenium(II) complexes are expected to be developed into a class of high-activity, low-toxicity antibacterial drugs with novel antimicrobial mechanisms.
